# An internet-based self-administered intervention for promoting healthy habits and weight loss in hypertensive people who are overweight or obese: a randomized controlled trial

**DOI:** 10.1186/s12872-015-0078-1

**Published:** 2015-08-04

**Authors:** Rosa M. Banos, Marinna S. Mensorio, Ausias Cebolla, Enrique Rodilla, Gonzalo Palomar, JuanFrancisco Lisón, Cristina Botella

**Affiliations:** Universitat de València, Facultad de Psicología, València, Spain; CIBER Fisiopatología Obesidad y Nutrición (CB06/03), Instituto Carlos III, Spain; CAPES Foundation, Ministry of Education of Brazil, Brasília, Brazil; Universitat Jaume I, Facultad de Psicología, Castelló de la Plana, Castelló, Spain; Hypertension and Vascular Risk Unit, Hospital de Sagunto, Sagunto, Spain; Universidad CEU Cardenal Herrera, Departamento de Medicina, Valencia, Spain; Primary Care Health Center, Quartell, Spain

**Keywords:** Lifestyle changes, Internet-based interventions, Unguided interventions, Hypertension, Obesity and overweight

## Abstract

**Background:**

The prevalence of overweight and obesity is on the rise worldwide with severe physical and psychosocial consequences. One of the most dangerous is hypertension. Lifestyle changes related to eating behaviour and physical activity are the critical components in the prevention and treatment of hypertension and obesity. Data indicates that the usual procedures to promote these healthy habits in health services are either insufficient or not efficient enough. Internet has been shown to be an effective tool for the implementation of lifestyle interventions based on this type of problem. This study aims to assess the efficacy of a totally self-administered online intervention programme *versus* the usual medical care for obese and overweight participants with hypertension (from the Spanish public health care system) to promote healthy lifestyles (eating behaviour and physical activity).

**Method:**

A randomized controlled trial will be conducted with 100 patients recruited from the hypertension unit of a public hospital. Participants will be randomly assigned to one of two conditions: a) SII: a self-administered Internet-based intervention protocol; and b) MUC-medical usual care. The online intervention is an Internet-delivered, multimedia, interactive, self-administered programme, composed of nine modules designed to promote healthy eating habits and increase physical activity. The first five modules will be activated at a rate of one per week, and access for modules 5 to 9 will open every two weeks. Patients will be assessed at four points: before the intervention, after the intervention (3 months), and at 6 and 12 months (follow-up). The outcome variables will include blood pressure, and Body Mass Index, as primary outcome measures, and quality of life and other lifestyle and anthropometrical variables as secondary outcome measures.

**Discussion:**

The literature highlights the need for more studies on the benefits of using the Internet to promote lifestyle interventions. This study aims to investigate the efficiency of a totally self-administered Internet − +based programme for promoting healthy habits and improving the medical indicators of a hypertensive and overweight population.

**Trial registration:**

NCT02445833

## Background

Lifestyle is one of the most important determinants of health. Since most communicable diseases are largely controlled in Western countries, current health problems are mainly related to behavioural variables [1], and most diseases are significantly influenced by lifestyle. Sedentary behaviour, consumption of fatty foods, fast foods, alcohol or tobacco, and stress, among other factors, are important causes of mortality and disease [2], and they have a determining influence on the maintenance of chronic diseases, including high blood pressure (hypertension) and obesity.

The World Health Organization [3] considers hypertension to be one of the most important public diseases in the world. This non-communicable disease is responsible for over a third of deaths from cardiac causes [4], and its costs are increasing alarmingly [5, 6]. The usual treatment for hypertension is based on medical-pharmacological interventions, however, evidence shows that changes in habits and lifestyles are crucial for treating this disease [3].

Obesity and being overweight (Body Mass Index: BMI ≥ 25 kg/m2) are also considered important health problems, due to their high prevalence, their multiple negative consequences, and their high costs [7, 8]. Although several factors are involved in their aetiology, evidence points to diet and physical activity as critical in both the prevention and treatment of obesity and associated disorders [9].

Hypertension and obesity are frequently associated. Overweight and obesity contribute to the occurrence of hypertension through different mechanisms, and weight loss is related to a drop in blood pressure. Evidence shows that blood pressure begins to drop in hypertensive obese patients they reach their ideal weight [10, 11].

Interventions in lifestyles are frequently recommended for both hypertension and obesity, however, these kinds of treatments collide with the problem of motivation, and environmental and personal barriers that often hinder their implementation. Non-adherence rates are usually very high for both obesity and hypertension [12], pointing out the need to develop alternative strategies to promote adherence and the success of lifestyle interventions. Although some interventions are effective in producing behavioural changes [13], these changes are generally moderate [14] and followed by a decline over time [15]. To improve outcomes in the medium and long term, there has been a proposal to adapt the interventions to the idiosyncratic characteristics of each person, emphasising the importance of providing constant and immediate feedback throughout the intervention [15]. Along these lines, and especially for chronic diseases, recent approaches to dispensing health services propose that these solutions should focus on the individual, providing information, services and tools that help them to implement and maintain behaviours related to health [16]. The objective is to replace previous models, focused on disease, with a more proactive approach to health, centred on welfare. This approach “empowers” the person by developing tools and services that people like, want, and can use, to achieve the necessary skills to respond appropriately in health-related situations [17].

To achieve these objectives, Information and Communication Technologies (ICTs) are a promising alternative, because they make it possible to provide personalised feedback [18] and they can be adapted flexibly to each user [19]. ICTs also have other important advantages, especially their good cost-benefit relationship and the possibility of increasing the efficiency of interventions, allowing them to reach a wider audience at a lower cost [20]. Interventions for hypertension and obesity could benefit from a shift in this direction, and there is evidence supporting the efficacy of online interventions for these conditions.

Regarding interventions for obese individuals, Levine et al. [21] reviewed the studies of technology-assisted weight loss interventions specifically provided in primary care settings, and the results showed that they helped patients achieve significant weight loss, giving participants the option to undergo the intervention semi-remotely. The authors concluded, however, that longer, pragmatic, interdisciplinary, open-source interventions are needed. In a similar way, meta-analyses performed by Neve et al. [22], Manzoni et al. [23], Kodama et al. [24], and Dutton et al. [25] found that Internet use in obesity treatment programmes has a modest but significant effect on weight control. These reviews also point out variations between trials (with smaller effect sizes, and inconsistent findings across studies), mixed results, heterogeneity of designs, and low generalization of the findings, however, and they emphasise that better descriptions of components of effective interventions are still needed. It is also necessary to develop and evaluate Internet-based weight-loss interventions that are specifically tailored to the needs of the health-care delivery system [23].

Regarding Internet-based lifestyle interventions for hypertension, studies that analyse and compare them with standard treatments are still scarce. In a recent systematic review, Vegting et al. [26] evaluated whether Internet-delivered care, as a complement to the usual care, improved cardiovascular outcomes. Nine studies were found, and only three specifically addressed hypertension [27–29]. Two of them found a significant decrease in BMI/weight [27, 29] and blood pressure (BP) measures [28, 29] in the intervention group compared to the control groups. More research is therefore needed in order to draw stronger conclusions about use of the internet in hypertension programmes.

In summary, Internet-based interventions are promising tools to promote a healthy lifestyle in people who are overweight or obese, and they can be of value in treating people who have hypertension and are at risk of developing metabolic syndrome. An intervention specifically for hypertensive obese patients, which is focused on changing their lifestyle, delivered over the Internet, and implemented in a public health context could be a cost-effective and affordable measure for reducing high blood pressure (BP).

The main objective of this study is to design, develop and test a completely self-administered Internet-based programme to promote healthy habits (diet and physical activity) in hypertensive patients with overweight or type I obesity who are at risk of developing diabetes mellitus. This programme will last three months, and it will be implemented in a public hospital service (a hypertension unit). The impact of this three-month programme will be analysed based on levels of BP, BMI, quality of life and other variables, and the results will be compared to a control group receiving their usual medical care.

## Method

### Study design

This is a two-armed, single centre, comparative, randomized controlled trial, comparing two conditions: a) a self-administered Internet-based intervention (SII) and b) usual medical care (UMC). The study will be conducted following the CONSORT statement (Consolidated Standards of Reporting Trials, http://www.consort-statement.org) [30, 31] and CONSORT-EHEALTH guidelines [32].

### Setting and study sample

The clinical trial will be conducted in the Hypertension and Vascular Risk Unit of the Hospital de Sagunto in Valencia (Spain). Participants will be recruited by physicians working in this unit. Participants should meet the following criteria: overweight or obese grade I (BMI > 25 and <35); age between 18–65 years; in clinical medical treatment for the prevention of metabolic syndrome or cardiac complications. Exclusion criteria are: no Internet access; taking more than three antihypertensive drugs; a diagnosis of diabetes; meeting the DSM-5 criteria for an eating disorder; having a disability that prevents or hinders exercise and physical activity; receiving any treatment for weight loss elsewhere.

### Sample size

The sample size for this project was based on the effect size found in previous studies [23, 33–35]. In previous RCT online interventions [33] a moderate effect size (g = 0.64) was found [23] when evaluating bodyweight. Taking this into account, a conservative effect size of .64 (Cohen’s d = .64) is expected in primary outcome measures. An estimation of the desired sample size was made based on the expected difference in the primary outcome variables, BP, BMI. Based on a power of 0.80 in a one-tailed test and an alpha of 0.05, 30 participants in each condition would be required. Considering the high levels (around 30 %) of dropout observed in this type of intervention [36], we chose to increase the expected sample to 50 participants in each condition. The hospital where the study took place selected a list of possible patients based on the inclusion and exclusion criteria.

### Recruitment and randomization

Participants will be recruited from the patient database of the hospital’s Hypertension Unit. The physicians in this unit will make a selection of patients who fit the inclusion criteria, then, a letter will be sent to these patients informing them of the study and requesting cooperation. Interested patients will be given an appointment at the hospital, where they will receive more detailed information. If patients agree to participate, they will be asked for their informed consent, and after signing, allocated to one of two groups: a) *Self-administered Internet-based Intervention (SII),* or *Usual Medical Care (UMC)*. Randomization will be performed using *random allocation software*. The programme Epidat 4.1 will be used to generate this sequence. The allocation will be carried out by an independent researcher, who will be unaware of the characteristics of the study. The sequence will be concealed until interventions are assigned. Patients will agree to participate before the random allocation and without knowing to which treatment they will be allocated. Study researchers conducting assessments across the whole study will be blind to participant treatment conditions and will be unaware of the group to which any patient belongs.

### Procedure

Participants will be given an appointment at the hospital in the first week to be assessed. They will then find out their randomization condition, and the following procedure will take place:

#### For participants in the SII condition

Patients will be given an explanation of the online intervention and instructions about how to access the modules, how to use them, and how to fulfill the online questionnaires. The modules will be active for three months during the intervention.

#### For participants in Usual Medical Care (UMC)

Patients will receive an explanation of the study, and how to fill out the questionnaires. Internet-based intervention will be offered to them at the end of the study (three months after its end) if they are interested. Patients will be called by their physicians following the usual care protocol.

### Internet-based intervention

The intervention programme is called “*Vivir mejor*” (“Living better”), and it is Internet-delivered (a web-page will be developed), multimedia, interactive, self-administered programme intervention composed of nine modules designed to gradually change participants’ eating habits and physical activity. The programme will be established as follows: the first five modules will be activated at a rate of one per week, and for modules 6 to 9, access will open every two weeks. The modules are:

#### M0. Welcome

Its purpose is to welcome participants to the programme, describe what it is and its structure (9 modules), explain the objectives and the main contents, and motivate participants to start the programme.

#### M1. Motivation for change

Its aim is to offer information about the importance of motivation in changing one’s lifestyle, and help participants understand that it is possible to increase motivation for change, know the costs and benefits of maintaining certain habits and behaviours and the costs and benefits of change, decide what relevant aspects of their day they should change, and learn to set specific and manageable goals to achieve the changes proposed.

#### M2. Nutrition and physical activity education

Its aim is to help participants to reflect on the benefits of eating well, obtain general information about the composition of foods, learn what physical activity is and differentiate it from exercise or sport, understand the role of physical activity in changing one’s lifestyle and weight loss, and have the initial keys to start being more active in everyday life.

#### M3. Strategies to overcome barriers

This aims to identify possible barriers and obstacles to healthy eating and physical activity, provide general information related to intake behaviours and learning little tricks, make participants aware of the importance of breakfast and helping them learn about the difference between different types of exercise, the recommended amount of physical activity, and the advantages and disadvantages of practicing at different times of the day, and making them reflect on the barriers that may be keeping them from starting to perform physical exercise and eating well.

#### M4. Setting goals and objectives in nutrition and physical activity, and modification of irrational beliefs

This aims to help participants identify the barriers that keep them from following the directions given to change their eating habits and activity, identify the role of thoughts in the choices and actions about food and physical activity, to develop self-knowledge strategies, learn the ABC technique, and learn other little tricks that will help them to eat more efficiently, and understand the importance of lunch and snack meals.

#### M5. Setting goals and objectives in nutrition and physical activity, and strategies for overcoming obstacles (emotional regulation, emotional eating and self-control)

This aims to help participants to identify what emotional eating is and discover how to cope with it, learn strategies to implement self-control in situations where compulsive eating behaviour can be observed, become aware of loss of control and learn the STOP! technique, and learn strategies to cope with situations/emotions that could lead to binge eating, as well as emotional regulation strategies.

#### M6. Strategies for overcoming obstacles and solving problems

Its aim is to give more information about the obstacles and barriers that often appear in the process of lifestyle change, and teach another coping strategy: the problem-solving technique.

#### M7. Intervention in body image difficulties and assertiveness

This is designed to help participants to understand the role of body image, identify concerns with their body image, work toward the development of a positive body image, and learn what assertiveness is and some techniques through which to practice it.

#### M8. Relapse prevention

This aims to summarise all the previous concepts and techniques, strengthen the changes made, set strategies to maintain the changes obtained, and prevent possible relapse.

#### Support during the intervention

This intervention is self-administered. A welcome e-mail will be sent when the participant starts the programme. Automatic e-mails will be sent after each module, encouraging the participant to continue to use the programme. To minimise the potential technological barriers that could hinder participation and adherence to the Internet-based intervention programme, patients will be provided with a contact email to answer technical questions or problems that might arise.

The instructions will recommend working on each module for a week. After each module, participants will receive positive feedback messages from the site. All modules include videos, texts, homework, and daily records. The participants can download the necessary files directly from the site.

**Fig. 1:**
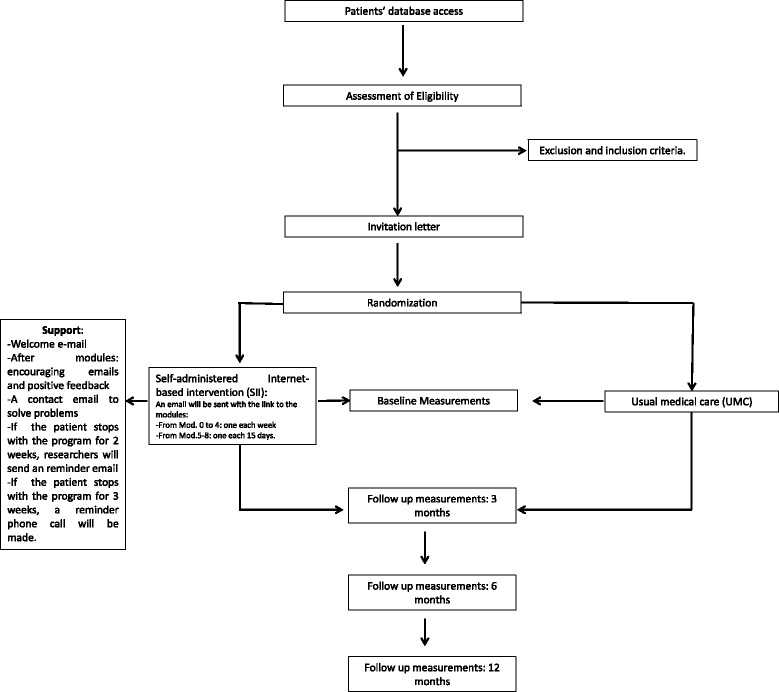
Study flow

Researchers will only contact participants if they stop accessing the modules for more than two weeks after they are posted. In this case, a reminder email will be sent. After three weeks without intervention access, a reminder telephone call will be made in order to ask the participants about any difficulties or doubts related to the use of the online protocol and help them solve any problems. The procedure details are shown in Fig. 1.

### Instruments

Participants in the SII condition will have four assessments: pre-intervention, post-intervention (3 months), Follow-up 1 (6 months), and Follow-up 2 (12 months). Measurements regarding satisfaction with the treatment will be filled out after each intervention module. Participants in the UCM condition will have two assessments: pre-intervention, post-intervention (3 months). At that time they will be invited to receive the Internet-based intervention. All the UCM participants who accept will be assessed again at 6 months (post-intervention), and Follow-up 1 (9 months) and Follow-up 2 (15 months). All the UCM participants who decided not to participant will be assessed at Follow-up 1 (6 months), and Follow-up 2 (12 months). The variables and assessment times are summarised in Table 1.

**Table 1:** Study variables and assessment points

Assessment area	Instrument	Time of assessment
Primary outcomes		
Blood pressure	Omron®	Baseline, post-T and follow-ups
BMI	TANITA	Baseline, post-T and follow-ups
Secondary outcome		
Gender, age	Sociodemographic question.	Baseline
Body fat and mass	TANITA	Baseline, post-T and follow-ups
Quality of life	QLI	Baseline, post-T and follow-ups
Other measures		
Physical activity	IPAQ	Baseline, post-T and follow-ups
Self-efficacy	GSES	Baseline, post-T and follow-ups
Intrinsic motivation	BREQ-2	Baseline, post-T and follow-ups
Treatment expectations	Treatment expectations	Baseline
Treatment satisfaction	Treatment satisfaction	Post-T

Follow-up sessions: Post-treatment, 6 and 12 months follow-ups

### Primary outcomes

The primary outcomes will be the blood pressure levels (arterial brachial systolic and diastolic pressure, determined by Omron®; in millimetres of mercury) and the BMI (Body Mass Index = kilogrammes/height ^2^(meters).

#### Secondary outcomes

The following variables will also be assessed:

- Body fat and body mass percentages, determined by TANITA, a bioimpedance scale.
- *Quality of life-QLI* [37, 38]: This includes 10 items, with a multiple-choice Likert response format on a scale from 1 to 10. It evaluates ten areas: physical, psychological, self-care and independent functioning welfare, occupational functioning, interpersonal functioning, social-emotional support, community and support services, personal fulfillment, spiritual fulfillment, overall perception of quality of life.
- *International Physical Activity Questionnaire - IPAQ - short* [39]: Self-report containing seven items that collect information about physical activity (PA) and sedentary behaviour in four areas: activity at work, when traveling, at home and during leisure time (leisure). The IPAQ addresses the number of days and minutes spent performing physical activities such as leisure-time occupations, locomotion and housework activities. The score is derived from the number of days, hours and minutes employed in these activities [40].

To assess treatment moderators, the following variables will also be measured:

- *GSES-12 Self-Efficacy Questionnaire General* [41, 42]: A self-report scale consisting of 10 items with a 4-point Likert scale. It was designed to assess overall efficacy and it evaluates the stable sense of personal competence to effectively manage a variety of stressful situations.
- *BREQ-2 - Behavioural Regulation in physical exercise and eating habits questionnaire* [43, 44]: The original questionnaire was developed to measure external regulation, introjected, identified and intrinsic motivation. The BREQ-2 scale consists of 19 items, compared to 15 on the original scale, which measure stages of the self-determination continuum on a 5-point Likert scale ranging from 0 (not true for me) to 4 (completely true for me), and adds the motivation factor. An adaptation of this scale can also be used to assess motivation to change eating habits.
- *Expectations about treatment* -adapted from Borkovec & Nau [45]: 6-item scale that uses a 10-point Likert scale. It evaluates user expectations about the online programme they will receive.
- *Satisfaction with treatment -* adapted from Borkovec & Nau [45]: 6-item scale with a 10-point Likert scale. It evaluates the user’s opinion about the programme received in terms of satisfaction and usefulness.

### Statistical data analysis

SPSS v.20 will be used for statistical analysis. Relevant statistical analyses will first be performed to verify proper randomization (independent samples *t* test for equivalence of groups). Intention-to-treat and per-protocol analysis will be used. Descriptive statistics for the variables included (mean and 95% confidence interval for normally distributed quantitative variables; and median and interquartile range for abnormally distributed quantitative variables) will be performed. A series of repeated-measures mixed ANOVAs with planned contrasts (2×2) will be carried out: with intergroup variable (condition - OI × UMC) × intragroup variable (time - pre- and post-) for each dependent variable. More sophisticated multivariate analysis, including multilevel regression, will be used. The effect size of improvement and intention-to-treat (ITT) will be estimated.

### Ethical issues

This project has been approved by the Ethics Committee for Clinical Research at the Hospital de Sagunto (ref: 002-2014-10) and by the Ministry of Health, Social Services and Equality in the Spanish Government (s/n), as a post-authorisation observational study. All participants will be informed of the study characteristics, and data will be treated with complete confidentiality. The requirements of the Organic Law on Data Protection (Law 15/1999 of December 13) will be followed at all times.

Participants will receive an Informed Consent document to sign before knowing which group they are in, but after receiving a general explanation of the study. Participants will also be informed about the completely voluntary nature of the research. For ethical reasons, patients allocated to the control group will have the opportunity to complete the online programme after the data collection.

## Discussion

Overweight and obesity are considered chronic conditions and their relationship as a promoter and potentially aggravating factor of hypertension is clearly recognised [46]. Hypertensive patients generally have unhealthy and sedentary habits. Universal non-targeted lifestyle interventions for weight loss are not sufficient for this population because they have specific demands and health conditions, in addition to health risks and medical treatments, that differ from other obese populations [26].

Studies assessing the efficacy of online interventions to change lifestyles in this hypertensive population are still scarce. From a public health perspective, it is necessary to develop programmes that promote healthy lifestyles in these patients and prevent the early stages of obesity before new diseases appear and increase the likelihood of cardiac complication associated with excess body weight [26, 47].

This study will be applied in the hypertension unit of a public hospital. Among the benefits of using the programme “Vivir Mejor” (Living Better), we would like to highlight: (a) it is a completely self-guided programme; (b) with the advantage of empowering patients, increasing their perception of self-efficacy, and making the patient the promoter and the person responsible for changing their lifestyles; (c) a low associated cost, as it does not need the supervision of clinical staff; (d) the potential to reach many people, with no time or geographical restrictions; (e) the possibility of continuously monitoring a patient’s progress during treatment without physical visits and (f) it introduces innovative technologies and treatments to aid health professionals in their daily work in public health contexts. Thus, with only a computer, patients will have access to an intervention to produce changes in lifestyle, with a focus on the two pillars indicated by the WHO: diet and physical activity.

The possible limitations of this study are: (a) a bias in the samples, as participants with no Internet access are excluded, and (b) it is not a multicentre study, as the patients will be selected just from one health centre.

The results of this project will allow researchers to continue to expand this programme, with improved versions that will include other important lifestyle elements that also play a very important role in chronic conditions like hypertension, such as alcohol, tobacco, stress management, sleep improvement. Comprehensive programmes can be established that address the lifestyle problem holistically and from different angles.

In conclusion, we have developed a protocol to evaluate whether an easy-to-use, self-administered, low-cost Internet-based programme can improve lifestyle, reduce hypertensive risks and produce healthy habits in hypertensive obese patients. Other studies have shown that these interventions can be effective in chronic disease populations. We expect that the present study will provide a starting point for developing simple and cost-effective interventions for reducing high BP, a condition that has serious health implications for society and individuals.

### Trial status

The trial is ongoing.

## References

[1] Straub RO, Straub RO (2007). Nutrition, obesity, and eating disorders. Health psychology: A biopsychosocial approach.

[2] Lowry R, Wechsler H, Galuska DA, Fulton JE, Kann L (2002). Television viewing and its associations with overweight, sedentary lifestyle, and insufficient consumption of fruits and vegetables among US high school students: differences by race, ethnicity, and gender. J Sch Health.

[3] WHO. Obesity and overweight. Geneva. 2013a. http://www.who.int/mediacentre/factsheets/fs311/en/index.html . Accessed 30 ene 2014.

[4] Arredondo A, Avilés R (2014). Hypertension and its effects on the economy of the health system for patients and society: suggestions for developing countries. Am J Hypertens.

[5] Mennini FS, Marcellusi A, von der Schulenburg JM, Gray A, Levy P, Sciattella P (2015). Cost of poor adherence to anti-hypertensive therapy in five European countries. Eur J Health Econ.

[6] Mozzafarian D, Benjamin EJ, Go AS, Arnett DK; Blaha MJ, Cushman M, et al. Heart disease and stroke statistics-2015 update: a report from the American Heart Association. Circulation. 2015;131:e29–322. 10.1161/CIR.0000000000000152 25520374

[7] WHO. A global brief on Hypertension. Gevena. 2013b. http://apps.who.int/iris/bitstream/10665/79059/1/WHO_DCO_WHD_2013.2_eng.pdf. Accessed 10 mar 2015.

[8] Padula WV, Allen RR, Nair KV (2014). Determining the cost of obesity and its common comorbidities from a commercial claims database. Clinical Obesity.

[9] Wilborn C, Beckham J, Campbell B, Harvey T, Galbreath M, La Bounty P (2005). Obesity: prevalence, theories, medical consequences, management, and research directions. J Int Soc Sports Nut.

[10] Re RN (2009). Obesity-related hypertension. Ochsber Journal.

[11] Kotchen TA (2008). Obesity-related hypertension? Weighing the evidence. Hypertension.

[12] Uzun S, Kara B, Yokuşoğlu M, Arslan F, Yılmaz MB, Karaeren H (2009). The assessment of adherence of hypertensive individuals to treatment and lifestyle change recommendations. Anadolu Kardiyol Derg.

[13] Kahn EB, Ramsey LT, Brownson RC, Heath GW, Howze EH, Powell KE (2002). The effectiveness of interventions to increase physical activity: a systematic review. Am J Prev Med.

[14] Foster C, Hillsdon M, Thorogood M. Interventions for promoting physical activity. Cochrane Database Systematic Review. 2005;1. doi:10.1002/14651858.CD003180.pub2. 10.1002/14651858.CD003180.pub2 PMC4164373 15674903

[15] Müller-Riemenschneider F, Reinhold T, Nocon M, Willich SN (2008). Long-term effectiveness of interventions promoting physical activity: a systematic review. Prev Med.

[16] Tengland PA (2012). Behavior change or empowerment: on the ethics of health-promotion strategies. Public Health Ethics.

[17] Saranummi N, Spruijt-Metz D, Intille SS, Korhone I, Nilsen WJ, Pavel M (2013). Moving the science of behavior change into the 21st century: novel solutions to prevent disease and promote health. IEEE Pulse.

[18] Norman GJ, Zabinski MF, Adams MA, Rosenberg DE, Yaroch AL, Atienza AA (2007). A review of eHealth interventions for physical activity and dietary behavior change. Am J Prev Med.

[19] Napolitano MA, Marcus BH (2002). Targeting and tailoring physical activity information using print and information technologies. Exerc Sport Sci Rev.

[20] Marcus BH, Nigg CR, Riebe D, Forsyth LH (2000). Interactive communication strategies: implications for population-based physical-activity promotion. Am J Prev Med.

[21] Levine DM, Savarimuthu S, Squires A, Nicholson J, Jay M (2015). Technology-assisted weight loss interventions in primary care: a systematic review. J Gen Intern Med.

[22] Neve M, Morgan PJ, Jones PR, Collins CE (2010). Effectiveness of web-based interventions in achieving weight loss and weight loss maintenance in overweight and obese adults: a systematic review with meta-analysis. Obes Rev.

[23] Manzoni GM, Pagnini F, Corti S, Molinari E, Castelnuovo G (2011). Internet-based behavioral interventions for obesity: an updated systematic review. Clin Pract Epidemiol Ment Health.

[24] Kodama S, Saito K, Tanaka S, Horikawa C, Fujiwara K, Hirasawa R (2012). Effect of web-based lifestyle modification on weight control: a meta-analysis. Int J Obes (Lond).

[25] Dutton GR, Laitner MH, Perri MG (2014). Lifestyle interventions for cardiovascular disease risk reduction: a systematic review of the effects of diet composition, food provision, and treatment modality on weight loss. Curr Atheroscler Rep.

[26] Vegting IL, Schrijver EJM, Otten RHJ, Nanayakkara PWB (2012). Internet programs targeting multiple lifestyle interventions in primary and secondary care are not superior to usual care alone in improving cardiovascular risk profile: a systematic review. Eur J of Intern Med.

[27] Bennett GG, Herring SJ, Puleo E, Stein EK, Emmons KM, Gillman MW (2010). Web-based weight loss in primary care: a randomized controlled trial. Obesity (Silver Spring).

[28] Green BB, Cook AJ, Ralston JD, Fishman PA, Catz SL, Carlson J (2008). Effectiveness of home blood pressure monitoring, Web communication, and pharmacist care on hypertension control: a randomized controlled trial. JAMA.

[29] Park MJ, Kim HS, Kim KS (2009). Cellular phone and internet-based individual intervention on blood pressure and obesity in obese patients with hypertension. Int J Med Inform.

[30] Moher D, Schulz KF, Altman DG (2001). The CONSORT statement: revised recommendations for improving the quality of reports of parallel-group randomized trials. J Am Pediatr Med Assoc.

[31] Moher D, Hopewell S, Schulz KF, Montori V, Gøtzsche PC, Devereaux PJ (2010). CONSORT 2010 Explanation and Elaboration: updated guidelines for reporting parallel group randomised trials. J Clin Epidemiol.

[32] Eysenbach G, CONSORT-EHEALTH Group (2011). CONSORT-EHEALTH: improving and standardizing evaluation reports of web-based and mobile health interventions. J Med Internet Res.

[33] Womble LG, Wadden TA, McGuckin BG, Sargent SL, Rothman RA, Krauthamer-Ewing ES (2004). A randomized controlled trial of a commercial internet weight loss program. Obes Res.

[34] McConnon A, Kirk SF, Cockroft JE, Harvey EL, Greenwood DC, Thomas JD (2007). The internet for weight control in an obese sample: results of a randomised controlled trial. BMC Health Serv Res.

[35] Carr LJ, Bartee RT, Dorozynski C, Broomfield JF, Smith ML, Smith DT (2008). Internet-delivered behavior change program increases physical activity and improves cardiometabolic disease risk factors in sedentary adults: results of a randomized controlled trial. Prev Med.

[36] Neve MJ, Collins CE, Morgan PJ (2010). Dropout, nonusage attrition, and pretreatment predictors of nonusage attrition in a commercial web-based weight loss program. J Med Internet Res.

[37] Mezzich JE, Cohen NL, Ruiperez MA. A quality of Life Index: brief description and validation. In: Paper presented at the International Congress of the International Federation for Psychiatric Epidemiology. Santiago de Compostela: Spain; 1996.

[38] Mezzich JE, Ruipérez MA, Pérez C, Yoon G, Liu J, Mahmud S (2000). The Spanish version of the quality of life index: presentation and validation. J Nerv Ment Dis.

[39] Booth ML (2000). Assessment of physical activity: an international perspective. Res Q Exerc Sport.

[40] Carral JMJ, Pérez CA (2011). Prevalencia y relación entre el nivel de actividad física y las actitudes alimenticias anómalas en estudiantes universitarias españolas de ciencias de la salud y la educación. Rev Esp Salud Pública.

[41] Baessler J, Schwarcer R (1996). Evaluación de la autoeficacia: adaptación española de la escala de Autoeficacia General. Ansiedad y Estrés.

[42] Markland D, Tobin V (2004). A modification to behavioural regulation in exercise questionnaire to include an assessment of a motivation. J Sport Exerc Psychol.

[43] Herrero R, Espinoza M, Molinari G, Etchemendy E, Garcia-Palacios A, Botella C (2014). Psychometric properties of the general self efficacy-12 scale in spanish: general and clinical population samples. Compr Psychiatry.

[44] Moreno JA, Cervelló E, Martínez A (2006). Measuring self-determination motivation in a physical fitness setting; validation of the Behavioural Regulation in Exercise Questionnaire-2 (BREQ-2) in a Spanish sample. J Sports Med Phys Fitness.

[45] Nguyen T, Lau DC (2012). The obesity epidemic and its impact on hypertension. Can J Cardiol.

[46] Borkovec TD, Nau SD (1972). Credibility of analogue therapy rationales. J Behav Ther Exp Psy.

[47] Liu S, Dunford SD, Leung YW, Brooks D, Thomas SG, Eysenbach G (2013). Reducing blood pressure with internet-based interventions: a meta-analysis. Can J Cardiol.

